# The antioxidant icariin protects porcine oocytes from age-related damage *in vitro*

**DOI:** 10.5713/ajas.20.0046

**Published:** 2020-05-12

**Authors:** Jae-Wook Yoon, Seung-Eun Lee, Yun-Gwi Park, Won-Jae Kim, Hyo-Jin Park, Chan-Oh Park, So-Hee Kim, Seung-Hwan Oh, Do-Geon Lee, Da-Bin Pyeon, Eun-Young Kim, Se-Pill Park

**Affiliations:** 1 Faculty of Biotechnology, College of Applied Life Sciences, Jeju National University, Jeju 63243, Korea; 2 Stem Cell Research Center, Jeju National University, Jeju 63243, Korea; 3 Mirae Cell Bio, Seoul 04795, Korea

**Keywords:** *In vitro* Aging, Porcine, Oocyte, Icariin, Antioxidant

## Abstract

**Objective:**

If fertilization does not occur within a specific period, the quality of unfertilized oocytes in the oviduct (*in vivo* aging) or in culture (*in vitro* aging) will deteriorate over time. Icariin (ICA), found in all species of *Epimedium* herbs, has strong antioxidant activity, and is thought to exert anti-aging effects *in vitro*. We asked whether ICA protects oocytes against age-related changes *in vitro*.

**Methods:**

We analyzed the reactive oxygen species (ROS) levels and expression of antioxidant, maternal, and estrogen receptor genes, and along with spindle morphology, and the developmental competence and quality of embryos in the presence and absence of ICA.

**Results:**

Treatment with 5 μM ICA (ICA-5) led to a significant reduction in ROS activity, but increased mRNA expression of glutathione and antioxidant genes (superoxide dismutase 1 [*SOD1*], *SOD2*, peroxiredoxin 5, and nuclear factor erythroid 2-like 2), during aging *in vitro*. In addition, ICA-5 prevented defects in spindle formation and chromosomal alignment, and increased mRNA expression of cytoplasmic maturation factor genes (bone morphogenetic protein 15, cyclin B1, MOS proto-oncogene, serine/threonine kinase, and growth differentiation factor-9). It also prevented apoptosis, increased mRNA expression of anti-apoptotic genes (BCL2-like 1 and baculoviral IAP repeat-containing 5), and reduced mRNA expression of pro-apoptotic genes (BCL2 antagonist/killer 1 and activation of caspase-3). Although the maturation and cleavage rates were similar in all groups, the total cell number per blastocyst and the percentage of apoptotic cells at the blastocyst stage were higher and lower, respectively, in the control and ICA-5 groups than in the aging group.

**Conclusion:**

ICA protects oocytes against damage during aging *in vitro*; therefore, it can be used to improve assisted reproductive technologies.

## INTRODUCTION

*In vitro* production technologies of embryos comprise three major successive stages: *in vitro* maturation (IVM) of immature oocytes, *in vitro* fertilization (IVF), and *in vitro* culture (IVC) of fertilized oocytes. The quality of mature oocytes *in vitro* determines the success of assisted reproductive technologies in mammalian species. In porcine *in vitro* production systems, immature oocytes are usually obtained from antral follicles (measuring 2 to 8 mm in diameter) in ovaries collected from a local slaughterhouse. Meiosis resumes spontaneously during culture of immature mammalian oocytes following removal from follicles. These oocytes undergo germinal vesicle breakdown after 16 to 20 h and reach metaphase of the second meiotic division (MII) by 40 h; then, meiosis arrests again until fertilization occurs. The nuclear and cytoplasmic events that occur during this process are referred to collectively as maturation and are required for monospermic fertilization and early embryonic development [1]. If fertilization does not occur within a specific period of time, the quality of unfertilized oocytes in the oviduct (*in vivo* aging) or in culture (*in vitro* aging) will deteriorate over time. The aging of oocytes is one of the factors limiting the results of various assisted reproductive technology in several mammalian species [2]. Several studies report that post-ovulatory aging correlates strongly with various oocyte defects, including zona pellucida hardening, spindle and chromosomal abnormalities, reduced capability for fertilization, abnormal development of embryos and fetuses, mitochondrial alterations, and changes in gene and protein expression [3,4]. In addition, post-ovulatory aging is accompanied by varied molecular, cellular, and biochemical changes, including mitochondria dysfunction, production of reactive oxygen species (ROS), decreased activity of maturation-promoting factors, decreased expression of the anti-apoptotic factor B-cell lymphoma 2 (*BCL-2*), activation of caspase-3 (*CASP3*), and changes in epigenetic modifications [2]. These deleterious aging-induced changes can reduce the quality of oocyte and adversely affect fertilization, and subsequent development of embryo. Therefore, many researchers have sought to develop methods that protect oocytes against *in vitro* aging.

The flavonoid icariin (ICA) is present in all species of *Epimedium* herbs and is extracted from the stem and leaves of the traditional Chinese medicinal plant *Epimedium* brevicornum Maxim (Herba Epimedii; family Berberidacae). ICA has a broad range of biological and pharmacological properties properties, including antioxidant and anti-inflammatory [5]. In addition, other studies have suggested that ICA acts as a phytoestrogen involved in activation of the estrogen receptor signaling pathway or cooperates with the estrogen-estrogen receptor complex in the nucleus [6].

We investigated the antioxidant effects of various concen trations of ICA during aging of porcine oocytes *in vitro*. We analyzed spindle morphology, levels of ROS, expression of antioxidant, estrogen receptor, and maternal genes in aged porcine oocytes treated with or without ICA. It also determined the developmental capacity and quality of embryos produced through parthenogenesis of these oocytes. The results show that ICA protects porcine oocytes against damage during aging *in vitro* by preventing oxidative stress. These findings may be applicable to aging during the IVM and help to protect oocytes against aging.

## MATERIALS AND METHODS

### Chemicals and reagents

All chemicals and reagents were purchased from Sigma (St. Louis, MO, USA) unless stated otherwise.

### Aging and *in vitro* maturation of porcine oocytes

Prepubertal porcine ovaries were collected from a local slaughterhouse and transported (within 2 h) at 30°C to 33°C to the laboratory in saline supplemented with 75 μg/mL penicillin G and 50 μg/mL streptomycin sulfate. Cumulus-oocyte complexes (COCs) were aspirated from follicles with a diameter of 2 to 8 mm using an 18-gauge needle and a disposable 10 mL syringe. COCs were washed three times in tissue culture medium (TCM)-199–HEPES containing 0.1% (w/v) bovine serum albumin (BSA). Thereafter, COCs (groups of 50 to 60) were matured in 500 μL TCM-199 (Gibco, Grand Island, NY, USA) containing Earle’s salts, 0.57 mM cysteine, 10 ng/mL epidermal growth factor, 0.5 μg/mL follicle-stimulating hormone, 0.5 μg/mL luteinizing hormone, and 10% (v/v) porcine follicular fluid under mineral oil for 44 h (control) at 38.8°C/5% CO_2_ in air. Oocyte aging was induced by culturing COCs for an additional 24 h (total of 68 h) (0, 5, 50, or 500 μM ICA) in TCM-199.

### Oocyte aging and icariin treatment

Mature oocytes were covered with mineral oil and cultured at 38.8°C in a humidified atmosphere of 5% CO_2_ in air in a 4-well dish containing 500 μL TCM-199. After maturation, MII oocytes were transferred to TCM-199 containing 0, 5, 50, or 500 μM ICA and cultured for an additional 24 h (total of 68 h) as described above. After treatment, oocytes were collected and aging was assessed.

### Parthenogenetic activation and embryo culture

We conducted parthenogenesis rather than IVF or intracytoplasmic sperm injection (ICSI). Before implantation, parthenogenesis can reveal the outcome (roughly) of IVF or ICSI. The results are not exact, but an approximate pattern can be determined.

Porcine oocytes mature after 44 h (control) or for an ad ditional 24 h (68 h total) (0, 5, 50, or 500 μM ICA), cumulus cells were removed by pipetting for 2 to 3 min in the presence of 1 mg/mL hyaluronidase. Parthenogenetic activation (PA) was induced by treating oocytes for 5 min with porcine zygote medium-5 containing 0.4% (w/v) BSA (IVC medium) and 5 μM Ca^2+^ ionomycin. After 3 h of culture in IVC medium containing 7.5 μg/mL cytochalasin B, embryos were washed three times in the IVC medium and cultured for 7 days at 38.8°C in a humidified atmosphere of 5% CO_2_ and 95% air. Oocytes and embryos were washed in Dulbecco’s phosphate-buffered saline (DPBS) and either fixed in 3.7% (w/v) paraformaldehyde for 20 min and stored at 4°C, or snap-frozen in liquid nitrogen and stored at −80°C, depending on the experiment.

### Measurement of intracellular reactive oxygen species and glutathione levels

Dichlorohydrofluorescein diacetate (DCFHDA) and CellTracker Blue 4-chloromethyl-6,8-difluoro-7-hydroxycoumarin (CMF_2_HC) were used to determine the intracellular levels of ROS and glutathione (GSH), respectively, as previously described [7,8], with slight modifications. Briefly, porcine oocytes mature after 44 h (control) or for an additional 24 h (68 h total) (0 and 5 μM ICA), cumulus cells were removed from COCs by pipetting in the presence of 0.1% (w/v) hyaluronidase. Denuded oocytes were incubated in the dark for 20 min at 38.8°C in DPBS containing 50 μM DCFHDA or 100 μM CMF_2_HC. Thereafter, oocytes were washed more than five times with DPBS containing 0.1% (w/v) BSA to completely remove excess dye and analyzed immediately by epifluorescence microscopy (Olympus, Tokyo, Japan). The ROS level was measured at excitation and emission wavelengths of 450 to 490 nm and 515 to 565 nm, respectively. The excitation and emission wavelengths of CMF_2_HC are 371 and 464 nm, respectively. Grayscale images were acquired with a digital camera (Nikon, Tokyo, Japan) attached to the microscope, and mean grayscale values were calculated using ImageJ software (NIH, Bethesda, MD, USA). Background fluorescence values were subtracted from the final values before statistical analysis. The replicate was repeated independently 6 to 7 times using 20 to 30 oocytes per experiment.

### Immunofluorescence analysis

Oocyte meiotic spindles and nuclei were visualized after maturation. Cumulus cells were removed from porcine COCs matured for 44 h (control) or an additional 24 h (total of 68 h) (0 and 5 μM ICA), and then oocytes were fixed overnight at 4°C in 4.0% (w/v) paraformaldehyde prepared in phosphate-buffered saline (PBS). Fixed oocytes were incubated in 0.5% (v/v) Triton X-100 for 30 min at 38.8°C. After blocking for 1 h with 1% BSA (w/v) prepared in PBS (blocking solution I), oocytes were incubated overnight at 4°C with a fluorescein isothiocyanate-conjugated anti-α-tubulin antibody (diluted 1:200 in blocking solution I). Nuclei were stained for 30 min with Hoechst 33342 (1 μg/mL). Finally, oocytes were washed three times with PBS containing 0.1% (w/v) BSA, mounted on glass slides, and observed under an inverted Olympus IX-71 microscope. To further investigate the effect of ICA on spindle organization, spindles without any abnormalities were classified as normal, whereas those in which chromosomes failed to align at the metaphase plate were classified as abnormal. Each experiment was repeated independently three times, and at least 20 oocytes were examined per group.

### Terminal deoxynucleotidyl transferase dUTP nick-end labeling and Hoechst staining

Porcine oocytes matured for 44 h (control) or an additional 24 h (total, 68 h) (0 and 5 μM ICA). At 7 days post-PA, blastocysts were fixed overnight at 4°C with 4.0% (w/v) paraformaldehyde prepared in PBS, washed more than three times with PBS containing 0.1% BSA, and then incubated at 38.8°C for 30 min with 0.1% Triton X-100. Blastocysts were incubated in the dark for 1 h at 38.8°C with fluorescein-conjugated dUTP and terminal deoxynucleotidyl transferase (*In Situ* Cell Death Detection Kit; Roche, Manheim, Germany). Mitotic and apoptotic cells were scored. Nuclei were stained for 30 min with Hoechst 33342 (1 μg/mL) and embryos were washed with PBS containing 0.1% BSA. Blastocysts were mounted on glass slides and examined under an inverted Olympus IX-71 fluorescence microscope. The experiment was repeated independently 3 to 4 times; at least 10 to 20 blastocysts were examined per group.

### Extraction of mRNA and synthesis of complementary DNA

First, mRNA was isolated from more than three biological replicates (30 to 40 oocytes per replicate) using a Dynabeads mRNA Direct Kit (Invitrogen, Carlsbad, CA, USA) according to the manufacturer’s instructions. Next, mRNA was collected in 10 μL elution buffer (provided with the kit). Eluted RNA was reverse-transcribed into complementary DNA using an oligo (dT) 20 primer and SuperScript II reverse transcriptase (Invitrogen, USA), according to the manufacturer’s instructions.

### Real-time reverse transcription polymerase chain reaction

The protocol used was the same as that described previously [9]. Real-time reverse transcription polymerase chain reaction (RT-PCR) was performed using the primer sets listed in Table 1 and a StepOnePlus Real-time PCR System (Applied Biosystems, Warrington, UK) in a final reaction volume of 20 μL containing SYBR Green PCR Master Mix (Applied Biosystems, UK). The PCR conditions were as follows: 10 min at 95°C, followed by 39 cycles of 15 s at 95°C and 60 s at 54°C or 60°C. Samples were then cooled to 12°C. Relative gene expression was analyzed using the 2^−ΔΔCt^ method [10] after normalization against expression of a housekeeping gene (maternal genes, estrogen receptor genes and apoptosis-related genes: glyceraldehyde-3-phosphate dehydrogenase; and antioxidant genes: β-actin). The experiment was repeated independently five times.

### Western blot analysis

The protocol was the same as that described previously [9]. In brief, oocytes (40 per sample) were solubilized in 20 μL of 1× sodium dodecyl sulfate (SDS) sample buffer (62.5 mM Tris-HCl, pH 6.8, containing 2% (w/v) SDS, 10% (v/v) glycerol, 50 μM dithiothreitol, and 0.01% (w/v) bromophenol blue or phenol red) and heated for 5 min at 95°C. Proteins were resolved for 1.5 h at 80 to 100 V on 5% to 12% Tris SDS-polyacrylamide gel electrophoresis gels. Samples were then transferred to Hybond-ECL nitrocellulose membranes (Amersham, Buckinghamshire, UK) at 300 mA for 2 h in transfer buffer (25 mM Tris, pH 8.5, containing 200 mM glycine and 20% [v/v] methanol). After blocking for 1 h with 5% (w/v) nonfat milk prepared in PBS, the membranes were incubated for at least 2 h with an anti-p44/42 MAPK or anti-phospho-p44/42 MAPK antibody diluted 1:500 in blocking solution (1× Tris-buffered saline, pH 7.5, containing 0.1% [v/v] Tween-20% and 5% [w/v] nonfat milk). Thereafter, the membranes were washed three times in TBST (20 mM Tris-HCl, pH 7.5, containing 250 mM NaCl and 0.1% [v/v] Tween-20) and incubated for 1 h with anti-rabbit immunoglobulin G-horseradish peroxidase diluted 1:2,000 in blocking solution. After three washes with TBST, immunoreactive protein bands were visualized in a dark room using X-ray films and a chemiluminescent luminol reagent (Invitrogen, USA). The amount of protein based on the band densities was calculated using ImageJ software (NIH, USA). The experiment was repeated independently three times.

### Statistical analysis

The general linear model procedure within the Statistical Analysis System (SAS User’s Guide, 1985, Statistical Analysis System Inc., Cary, NC, USA) was used to analyze data from all experiments. The paired Tukey’s multiple range test were used to determine significant differences. Statistical significance is defined when p values are less than 0.05.

## RESULTS

### Icariin enhances the embryo development of aging porcine oocytes *in vitro*

Porcine oocytes were matured *in vitro* for 44 h (control) or an additional 24 h (total, 68 h) in the presence of 0, 5, 50, or 500 μM ICA (referred to as aging, ICA-5, ICA-50, and ICA-500, respectively) to determine the optimal concentration. There was no difference between the groups in the rate of MII reach of porcine oocytes. The percentage of oocytes that reached the 2 to 4-cell stage through cleavage after PA did not differ between the aging, ICA-5, ICA-50, ICA-500, and control groups (Table 2). The rate of blastocyst development at day 7 of cleaved oocytes was significantly higher (p<0.05) in the control and ICA-treated groups than in the aging group; the percentages between the ICA-5, ICA-50, and ICA-500 groups were similar (Table 2). Therefore, we compared the control, aging, and ICA-5 groups in subsequent experiments. The replicate was repeated seven independent times, with 50 to 60 oocytes per experiment.

### Icariin reduces the level of reactive oxygen species in aging porcine oocytes *in vitro*

**Figure 1:**
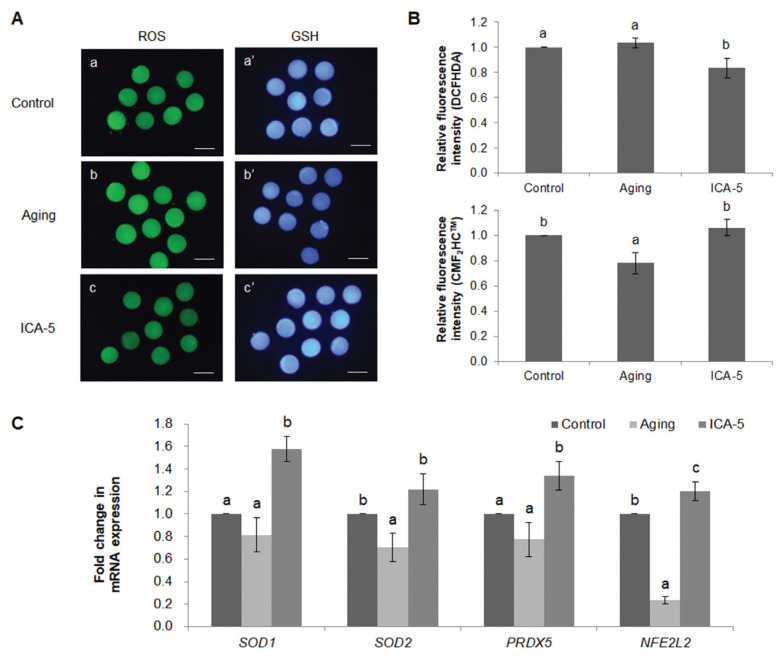
Antioxidant effect of icariin (ICA) during aging of porcine oocytes *in vitro*. (A) Epifluorescence images of oocytes stained with DCHFDA (green) and CellTracker Blue CMF_2_HC (blue). a and a′: control group; b and b′: aging group; and c and c′: ICA-5 group. a, b, and c: ROS staining; a′, b′, and c′: GSH staining. (B) Fluorescence intensity of intracellular ROS staining. (C) Fluorescence intensity of intracellular GSH staining. (D) Relative expression of antioxidant genes *SOD1*, *SOD2*, *PRDX5*, and *NFE2L2*. Data are derived from 3 to 4 independent replicates per group are expressed as the mean±standard error of the mean. ^a–c^ p<0.05. Scale bar = 120 μm. GSH, glutathione; ROS, reactive oxygen species; *SOD1*, superoxide dismutase 1; *SOD2*, superoxide dismutase 2; *PRDX5*, peroxiredoxin 5; *NFE2L2*, nuclear factor erythroid 2-like 2.

At the MII stage, the effects of ICA on ROS and GSH levels were analyzed by staining oocytes with DCFHDA and CMF_2_HC, respectively (Figure 1A). ROS levels in the ICA-5 group were significantly lower (p<0.05) than those in the aging group, but there was no difference between the control and aging groups (control, 1.0±0.0 pixels/oocyte; aging, 1.0±0.0 pixels/oocyte; and ICA-5, 0.8±0.1 pixels/oocyte; Figure 1B). The staining intensity of GSH was significantly higher (p<0.05) in the control and ICA-5 groups than in the aging group (control, 1.0±0.0 pixels/oocyte; aging, 0.8± 0.1 pixels/oocyte; and ICA-5, 1.1±0.1 pixels/oocyte; Figure 1C).

Expression of the antioxidant genes superoxide dismutase 1 (*SOD1*), superoxide dismutase 2 (*SOD2*), peroxiredoxin 5 (*PRDX5*), and nuclear factor erythroid 2-like 2 (*NFE2L2*) was analyzed by real-time RT-PCR (Figure 1D). mRNA expression of *SOD1* and *PRDX5* was significantly higher (p< 0.05) in the ICA-5 group than in the aging group, and was similar in the control and aging groups. mRNA expression of *SOD2* was significantly higher (p<0.05) in the ICA-5 group than in the aging group, but similar to that in the control group. mRNA expression of *NFE2L2* was significantly higher (p<0.05) in the ICA-5 group than in the aging and control groups.

### Icariin prevents chromosome misalignment and abnormal spindle organization in aging porcine oocytes *in vitro*

**Figure 2:**
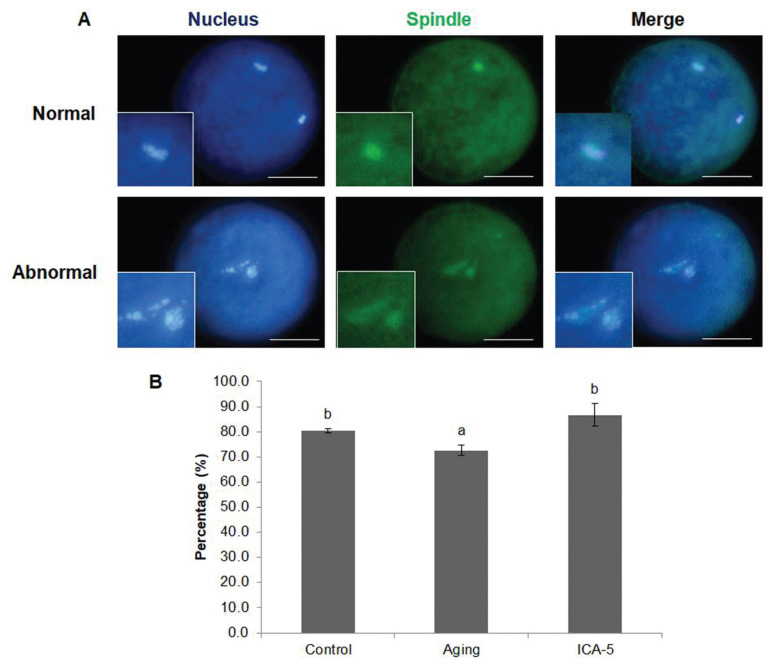
Effect of icariin (ICA) on meiotic spindle morphology in porcine oocytes during aging *in vitro*. (A) Normal and abnormal chromosome alignment and meiotic spindle formation in oocytes. (B) Percentage of oocytes in which the morphology of the chromosomes and the meiotic spindle were normal. Data are derived from three independent replicates per group and expressed as the mean±standard error of the mean (^a,b^ p<0.05). Scale bar = 50 μm.

The percentage of oocytes with normal meiotic spindles at stage MII was significantly higher (p<0.05) in the ICA-5 group than in the aging group, but was similar in the control and ICA-5 groups (control, 80.3%±0.8%; aging, 72.6%±2.1%; and ICA-5, 86.7%±4.5%; Figure 2).

### Icariin increases the expression of cytoplasmic maturation markers in porcine oocytes during aging *in vitro*

**Figure 3:**
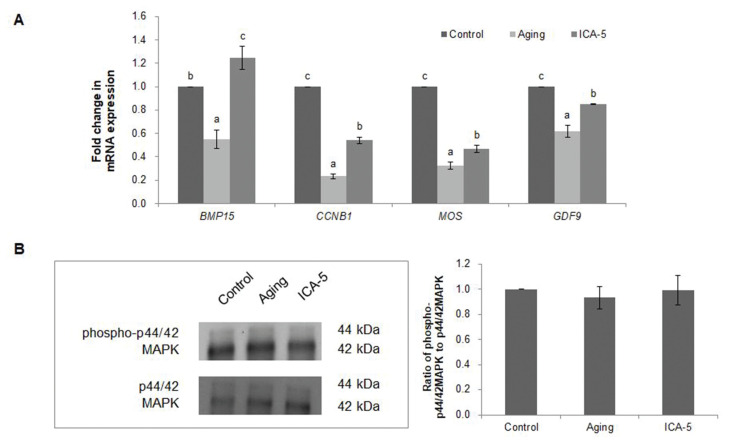
Effect of icariin (ICA) treatment of aging porcine oocytes on expression of maternal genes and MAPK activity. (A) Maternal gene expression. (B) MAPK activity. Data were normalized against levels in the control group. Data are derived from three or five independent replicates per group and are expressed as the mean±standard error of the mean (^a–c^ p<0.05).

In order to investigate the effect of ICA on cytoplasmic maturation of aging oocytes in the MII stage, the expression of maternal genes and MAPK activity werw examined (Figure 3). Expression of the cytoplasmic maturation marker genes bone morphogenetic protein 15 (*BMP15*), cyclin B1 (*CCNB1*), MOS proto-oncogene, serine/threonine kinase (*MOS*), and growth differentiation factor-9 (*GDF9*) was analyzed by real-time RT-PCR (Figure 3A). Expression of mRNA encoding *CCNB1*, *MOS*, and *GDF9* was significantly higher (p<0.05) in the ICA-5 group than in the aging group, but significantly lower (p<0.05) than in the control group. mRNA expression of *BMP15* was significantly higher (p<0.05) in the ICA-5 group than in the aging and control groups. Western blotting has shown that the active form of this kinase, phosphorylated p44/42 MAPK (phospho-p44/42 MAPK), migrated as a doublet in lysates of matured and aged porcine oocytes. The relative ratio of phospho-p44/42 MAPK to total p44/42 MAPK did not differ significantly (p<0.05) between the groups (control, 1.0±0.0; aging, 0.9±0.1; and ICA-5, 1.0±0.1; Figure 3B).

### Icariin increases the expression of estrogen receptor genes in porcine oocytes during aging *in vitro*

**Figure 4:**
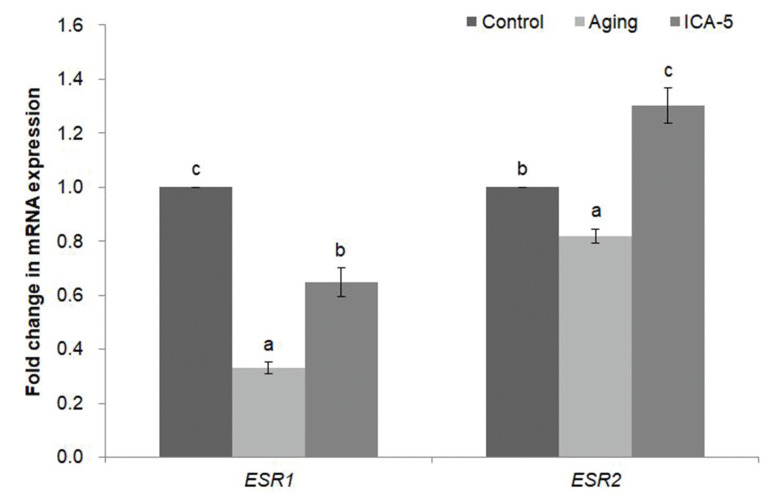
Effect of icariin (ICA) treatment of aging porcine oocytes on expression of estrogen receptor genes. Data are derived from five independent replicates per group and are expressed as the mean±standard error of the mean (^a–c^ p<0.05).

At the MII stage, the effects of ICA on mRNA expression of estrogen receptor 1 (*ESR1*) and 2 (*ESR2*) were analyzed by real-time RT-PCR to determine whether ICA activates these receptors. mRNA expression of *ESR1* was significantly higher (p<0.05) in the ICA-5 group than in the aging group, but it was significantly lower (p<0.05) than in the control group (Figure 4). mRNA expression of *ESR2* was significantly higher (p<0.05) in the ICA-5 group than in the control and aging groups (Figure 4).

### Icariin alters expression of apoptosis-related genes in porcine oocytes during aging *in vitro*

**Figure 5:**
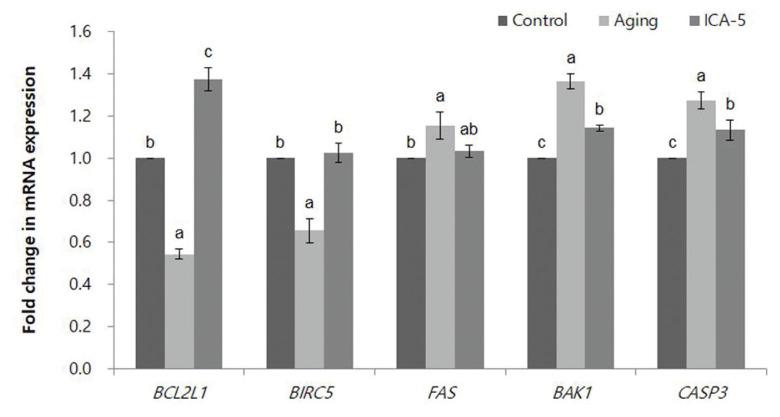
Effect of icariin (ICA) treatment of aging porcine oocytes on expression of apoptosis-related genes. Data are derived from five independent replicates per group and are expressed as the mean±standard error of the mean (^a–c^ p<0.05).

At the blastocyst stage, expression of apoptosis-related genes BCL2-like 1 (*BCL2L1*), baculoviral IAP repeat-containing 5 (*BIRC5*), Fas cell surface death receptor (*FAS*), BCL2 antagonist/killer 1 (*BAK1*), and *CASP3* was analyzed by real-time RT-PCR (Figure 5). mRNA expression of *BCL2L1* was significantly higher (p<0.05) in the ICA-5 group than in the control and aging groups, whereas expression of that *BIRC5* was significantly higher (p<0.05) in the ICA-5 group than in the aging group; however, it was similar in the control and ICA-5 groups. Expression of *FAS* was significantly higher (p<0.05) in the aging group than in the control group, was similar in the control and ICA-5 groups, and was not significantly different between the aging and ICA-5 groups. mRNA expression of *BAK1* and *CASP3* was significantly higher (p<0.05) in the ICA-5 group than in the control groups, but significantly lower (p<0.05) than in the aging group.

### Icariin improves the quality and developmental capacity of embryos derived from aged porcine oocytes *in vitro*

**Figure 6:**
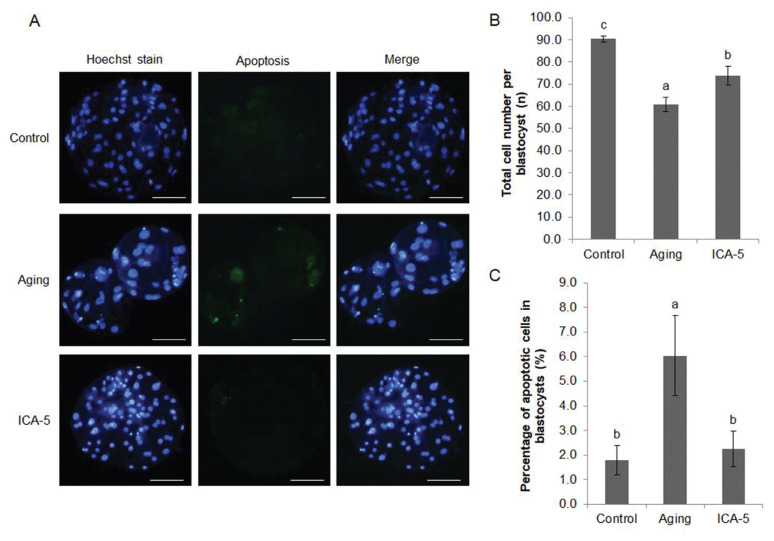
Effect of icariin (ICA) treatment of aging of porcine oocytes on subsequent embryo quality. (A) Blastocyst staining. (B) Total cell number per blastocyst. (C) Percentage of apoptotic cells within the blastocyst. Data are derived from 3 to 4 independent replicates per group and are expressed as the mean±standard error of the mean (^a–c^ p<0.05). Scale bar = 50 μm.

To investigate whether ICA treatment during IVM of oocytes affects subsequent embryo quality and development, we matured oocytes in the treated with or without of 5 μM ICA and then subjected them to PA. The total number of cells per blastocyst was significantly lower (p<0.05) in the ICA-5 group than in the control group. However, the number in the aging group was significantly lower (p<0.05) than that in the ICA-5 group (control, 90.4±1.4; aging, 60.9±3.3; and ICA-5, 73.8±4.2; Figure 6B). Fragmentation of genomic DNA was assessed by terminal deoxynucleotidyl transferase dUTP nick-end labeling to detect apoptotic cells within the blastocyst. The percentage of apoptotic cells in the aging group was significantly higher (p<0.05) than that in the control and ICA-5 groups (control, 1.8%±0.6%; aging, 6.0%±1.6%; and ICA-5, 2.3%±0.7%; Figure 6C).

## DISCUSSION

Oocytes are damaged during aging *in vitro*, and the mechanism by which aged oocytes are protected is unknown. This study investigated the effects of ICA, an antioxidant, on the damage that occurs during aging of porcine oocytes *in vitro*. The easiest way to evaluate the quality of oocytes *in vitro* is to calculate the rate of developmental. This determines the efficiency of the embryos produced *in vitro*. *In vitro* aging of oocytes adcersely affects embryo development and oocyte comperency, and reduces the rate of oocytes cleavage. Our data showed that the developmental rate in the aging group decreased; this was not the case and prevents deterioration of blastocyst quality in the 5 μM ICA treated group. This demonstrates that ICA improves embryonic developmental competence by protecting oocytes from age-related damage *in vitro*.

Previous studies show that ICA reversed ROS damage in duced by H_2_O_2_ treatment in mouse embryos [11]. In addition, ICA inhibits ROS production in lipopolysaccharide-treated microglia [12], and could inhibit H_2_O_2_-induced human umbilical vein endothelial injury [13]. Oxidative stress arises in post-ovulatory aging oocytes, in which ROS levels increase with a concomitant reduction in antioxidant protection [14]. Although intracellular GSH plays an important role in protecting oocytes from oxidative damage, levels fall gradually during aging [14]. Our results suggest that ICA decreases the level of ROS and prevents the decrease of GSH levels in oocytes during *in vitro* aging. *SOD1* converts two superoxide anions, which are normal products of cellular respiration, into hydrogen peroxide and oxygen [2O_2_^−^+2H^+^→H_2_O_2_+O_2_] [15]. *SOD2* reduces the superoxide anion produced as a byproduct of oxidative phosphorylation to generate hydrogen peroxide and oxygen [16]. *PRDX5* protects cells against ROS by prioritizing elimination of hydrogen peroxide and alkyl hydroperoxides [17]. *NFE2L2* transactivates genes containing antioxidant response elements and coordinates expression of cytoprotective genes to protect cells against oxidative stress [18]. Our result showed that expression of these antioxidant genes was increased the ICA-5 group. These results are supported by previous results that ICA decreases the level of ROS and inhibits the decrease of GSH level. Taken together, these results suggest that ICA protects oocytes against oxidative stress, thereby decreases the ROS level, inhibiting the decrease in GSH level, and increasing gene expression.

Chromosome condensation is the most noticeable event during meiotic maturation and is important for formation and proper separation of chromosomes. Oxidative stress generated during aging in the maturation stage of the oocyte has a negative effect on cytoplasmic maturation and nuclear maturation [14,19]. In previous studies, there was no data for ICA treatment on oocytes during *in vitro* aging. However, previous studies have shown that other antioxidants (hesperetin and allicin) protect oocytes against oxidative stress received during aging *in vitro* and inhibit abnormal spindle formation and decrease of maternal genes and MAPK activity [14,19]. Treatment with hesperetin during aging of oocytes *in vitro* has been shown to protect the chromosomes and spindles in MII and prevent the decrease in expression of the maternal genes *CCNB1*, *MOS*, *BMP15*, and *GDF9* [14]. In oocytes, transcription is mostly quiescent and gene expression is regulated by translational rather than transcriptional mechanism [20]. However, the oocytes of control group which completed maturation fully expressed maternal mRNA, whereas the mRNA expression of aged oocytes was downregulated compared to the oocytes of control group. Therefore, we confirmed the protection of maternal mRNA in aged oocytes treated with ICA and also confirmed the translation of mRNA through MAPK activation. Our data showed that the percentage of oocytes with normal spindles in the ICA-5 group was higher than that in the aging group. In additional, although expression of maternal genes increased in the ICA-5 group, phosphorylation of MAPK was no difference in the all groups. Taken together, these results suggest that ICA prevents deterioration of oocyte quality by maintaining nuclear maturation and maternal genes expression. In addition, these results indicate that ICA is a potent compound that helps maintain healthy oocytes by improving the conditions for IVC of oocytes.

ICA might act via estrogen receptors, or in cooperation with estrogen receptor signaling [6]. We examined expression of *ESR1* and *ESR2* to investigate whether ICA enters oocytes via estrogen receptors. The two types of estrogen receptor are ERα and ERβ, which are encoded by *ESR1* and *ESR2*, respectively. ERβ is homologous to ERα; indeed, these two proteins have similar, but not identical, tissue distributions. The expression of receptor genes fell in the aging group, and that ICA prevents this decrease. The data suggest that ICA enters porcine oocytes through estrogen receptors.

Apoptosis occurs during aging and development; it is also a maintain cell populations within tissues as a homeostasis mechanism [21]. In addition, apoptosis is a form of programmed cell death that kills individual cells while preserving the overall structure of the surrounding tissue [22]. Excessive apoptosis can affect blastocyst maturation, induce death of an early embryo, and cause fetal deformities [23]. Therefore, we monitored the expression levels of apoptosis-related genes in order to investigate the extent of apoptosis. Several genes control apoptosis. As a dominant inhibitor of apoptosis, *BCL2L1* is a central regulator of programmed cell death and an important target for anti-cancer drugs [24]. *BIRC5* belongs to the inhibitors of apoptosis family; as such, it is involved in regulating cell division and inhibiting apoptosis [25]. *BAK1*, a member of the BCL-2 family, is an important regulator of mitochondrial apoptosis [26]. Caspases, the primary drivers of apoptotic cell death, cleave cellular proteins, a process that is critical for dismantling dying cells [22]. *CASP3* is the most well-characterized effector caspase [22]. Specifically, *CASP3* is the executioner caspase and functions during the final phase of apoptosis. Consequently, it is cleaved and activated during late apoptotic events [27]. A previous study reported that ICA prevents apoptosis in human vascular endothelial cells following exposure to oxidized low-density lipoprotein by regulating expression of *BCL-2* and *CASP3* protein and mRNA [28]. It addition, ICA decreased the expression of pro-apoptotic protein (cleaved caspase3 and cleaved poly (ADP-ribose) polymerase), by inhibiting the apoptotic signaling pathway in K562 cell exposed to radiation [29]. These results indicate that ICA increased the expression of anti-apoptotic genes and decreased the expression of pro-apoptotic genes in aged oocytes, which suggested that ICA effectively blocks apoptosis during oocyte aging by regulating the expression of pro-apoptosis genes or anti-apoptosis genes.

In conclusion, this study indicates that treatment of aging oocytes with 5 μM ICA reduces the level of ROS, prevents decreased expression of antioxidant genes (effectively protecting oocytes against oxidative stress), and prevents decreased expression of maternal genes, thereby minimizing deterioration in oocyte quality during aging. Moreover, ICA prevents decreased expression of anti-apoptotic genes, thereby preventing increased expression of pro-apoptotic genes. Finally, ICA increases good-quality blastocyst production by supporting blastocyst development and formation. Thus, ICA may be useful for improving assisted reproductive technologies.

## Figures and Tables

**Table 1:** Primers used for real-time reverse transcription polymerase chain reaction

Gene	GenBank accession no.	Primer sequence	Annealing temperature (°C)	Product size (bp)
*GAPDH*	AF017079.1	F: GATGACATCAAGAAGGTGGT	54	100
		R: CACTGTTAAAGTCAGAGGACAC		
*β-actin*	AY550069.1	F:AGATCATGTTCGAGACCTTC	54	220
		R: GTCAGGATCTTCATGGGTAGT		
*GDF9*	XQ687750.1	F: GTCTCCAACAAGAGAGAGATTC	54	109
		R: CTGCCAGAAGAGTCATGTTAC		
*BMP15*	NM_001005155.2	F: GACACTGCCTTCTTGTTACTC	54	94
		R: CTCTTGCCATAAACTCTTCC		
*CCNB1*	NM_001170768.1	F: ATACCTACTGGGTCGTGAAG	54	97
		R: GGTCTCCTGTAGTAACCTGAAT		
*MOS*	NM_001113219.1	F: ACCTTACACCAGGTCATCTAC	54	105
		R: GGAATACTTGAGACACTTCTCC		
*SOD1*	GU944822.1	F: GTGTTAGTAACGGGAACCAT	54	120
		R: GGATTCAGGATTGAAGTGAG		
*SOD2*	NM_214127.2	F: AGACCTGATTACCTGAAAGC	54	110
		R: CTTGATGTACTCGGTGTGAG		
*PRDX5*	AF110735.2	F: GGCATGTCTGAGTGTTAATG	54	118
		R: ATCTGTCTCCTTCCCAAAG		
*NFE2L2*	Gu991000.1	F: CTATGGAGACACACTGCTTG	54	99
		R: ACAGGCTGTGTTTTAGGACT		
*ESR1*	NM_214220.1	F: TGGAGTGTACACGTTTCTGT	54	87
		R: GTGTCTGTGATCTTGTCCAG		
*ESR2*	NM_001001533.1	F: AACTCTCCTGTCTCCTACAACT	54	91
		R: GGCAGCTTTCTACATAGGAG		
*BCL2L1*	NM_214285.1	F: GGTTGACTTTCTCTCCTACAAG	54	118
		R: CTCAGTTCTGTTCTCTTCCAC		
*BIRC5*	NM_214141.1	F: CTTCTGCTTCAAAGAGCTG	54	154
		R: GGCTCTTTCTTTGTCCAGT		
*FAS*	AJ001202.1	F: GAGAGACAGAGGAAGACGAG	54	194
		R: CTGTTCAGCTGTATCTTTGG		
*BAK1*	AJ001204	F: CTAGAACCTAGCAGCACCAT	60	151
		R: CGATCTTGGTGAAGTACTC		
*CASP3*	NM_214131	F: GACTGCTGTAGAACTCTAACTGG	54	110
		R: ATGTCATCTTCAGTCCCACT		

F, forward; R, reverse; *GAPDH*, glyceraldehyde-3-phosphate dehydrogenase; *GDF9*, growth differentiation factor-9; *BMP1*5, bone morphogenetic protein 15; *CCNB1*, cyclin B1; *MOS*, MOS proto-oncogene, serine/threonine kinase; *SOD1*, superoxide dismutase 1; *SOD2*, superoxide dismutase 2; *PRDX5*, peroxiredoxin 5; *NFE2L2*, nuclear factor erythroid 2-like 2; *ESR1*, estrogen receptor 1; *ESR2*, estrogen receptor 2; *BCL2L1*, BCL2-like 1; *BIRC5*, baculoviral IAP repeat-containing 5; *FAS*, Fas cell surface death receptor; *BAK1*, BCL2 antagonist/killer 1; *CASP3*, caspase-3.

**Table 2:** Effect of icariin treatment on subsequent embryo development by porcine oocytes during aging *in vitro*

Treatment group	ICA concentration (μM)	No. of germinal vesicle oocytes	No. (%) of surviving oocytes1)	No. (%) of cleaved oocytes on day 22)	No. (%) of blastocysts on day 73)
Control	0	56	51 (91.4±1.6)	37 (72.8±5.6)	17 (45.8±3.2)^c^
Aging	0	56	51 (89.8±1.6)	41 (80.2±5.0)	9 (22.9±1.9)^a^
ICA-5	5	56	52 (92.0±0.8)	45 (86.0±4.7)	16 (34.8±2.3)^bc^
ICA-50	50	59	54 (91.5±2.9)	42 (77.7±3.7)	15 (36.8±4.8)^b^
ICA-500	500	59	53 (90.5±1.7)	42 (81.6±4.1)	16 (37.8±2.3)^b^

Values represent the mean±standard error of the mean of independent experiments.

ICA, icariin.

1) Percentage of oocytes that reached MII.

2) Percentage of oocytes that underwent cleavage.

3) Percentage of cleaved oocytes that reached the blastocyst stage on day 7.

a–c Values within the same column with different superscript letters are significantly different (p<0.05).
